# Lesion detection by [^89^Zr]Zr-DFO-girentuximab and [^18^F]FDG-PET/CT in patients with newly diagnosed metastatic renal cell carcinoma

**DOI:** 10.1007/s00259-019-04358-9

**Published:** 2019-06-06

**Authors:** Sarah R. Verhoeff, Suzanne C. van Es, Eline Boon, Erik van Helden, Lindsay Angus, Sjoerd G. Elias, Sjoukje F. Oosting, Erik H. Aarntzen, Adrienne H. Brouwers, Thomas C. Kwee, Sandra Heskamp, Otto S. Hoekstra, Henk Verheul, Astrid A. M. van der Veldt, Elisabeth G. E. de Vries, Otto C. Boerman, Winette T. A. van der Graaf, Wim J. G. Oyen, Carla M. L. van Herpen

**Affiliations:** 1 Department of Medical Oncology, Radboud University Medical Center Nijmegen, P.O. Box 9101, 6500 HB Nijmegen, The Netherlands; 2 Department of Medical Oncology, University of Groningen, University Medical Center Groningen, Groningen, The Netherlands; 3 Department of Medical Oncology, Cancer Center Amsterdam, Amsterdam, The Netherlands; 4 Departments of Medical Oncology and Radiology & Nuclear Medicine, Erasmus University Medical Center, Rotterdam, The Netherlands; 5 Department of Epidemiology, Julius Center for Health Sciences and Primary Care, University Medical Center Utrecht, Utrecht University, Utrecht, The Netherlands; 6 Department of Radiology and Nuclear Medicine, Radboud University Medical Center, Nijmegen, The Netherlands; 7 Department of Nuclear Medicine and Molecular Imaging, University of Groningen, University Medical Center Groningen, Groningen, The Netherlands; 8 Department of Radiology, University of Groningen, University Medical Center Groningen, Groningen, the Netherlands; 9 Department of Radiology and Nuclear Medicine, Amsterdam UMC, Vrije Universiteit Amsterdam, Cancer Center Amsterdam, Amsterdam, The Netherlands; 10 Department of Medical Oncology, Netherlands Cancer Institute, Amsterdam, The Netherlands; 11 Department of Radiology and Nuclear Medicine, Rijnstate, Arnhem, The Netherlands; 12 Department of Biomedical Sciences, Humanitas University, Milan, Italy

**Keywords:** CAIX, Clear cell renal cell carcinoma, FDG, Girentuximab, Imaging, PET

## Abstract

**Purpose:**

The main objective of this preliminary analysis of the IMaging PAtients for Cancer drug selecTion (IMPACT)-renal cell cancer (RCC) study is to evaluate the lesion detection of baseline contrast-enhanced CT, [^89^Zr]Zr-DFO-girentuximab-PET/CT and [^18^F]FDG-PET/CT in detecting ccRCC lesions in patients with a good or intermediate prognosis metastatic clear cell renal cell carcinoma (mccRCC) according to the International Metastatic Database Consortium (IMDC) risk model.

**Methods:**

Between February 2015 and March 2018, 42 newly diagnosed mccRCC patients with good or intermediate prognosis, eligible for watchful waiting, were included. Patients underwent CT, [^89^Zr]Zr-DFO-girentuximab-PET/CT and [^18^F]FDG-PET/CT at baseline. Scans were independently reviewed and lesions of ≥10 mm and lymph nodes of ≥15 mm at CT were analyzed. For lesions with [^89^Zr]Zr-DFO-girentuximab or [^18^F]FDG-uptake visually exceeding background uptake, maximum standardized uptake values (SUV_max_) were measured.

**Results:**

A total of 449 lesions were detected by ≥1 modality (median per patient: 7; ICR 4.25–12.75) of which 42% were in lung, 22% in lymph nodes and 10% in bone. Combined [^89^Zr]Zr-DFO-girentuximab-PET/CT and CT detected more lesions than CT alone: 91% (95%CI: 87–94) versus 56% (95%CI: 50–62*, p* = 0.001), respectively, and more than CT and [^18^F]FDG-PET/CT combined (84% (95%CI:79–88, *p* < 0.005). Both PET/CTs detected more bone and soft tissue lesions compared to CT alone.

**Conclusions:**

The addition of [^89^Zr]Zr-DFO-girentuximab-PET/CT and [^18^F]FDG-PET/CT to CT increases lesion detection compared to CT alone in newly diagnosed good and intermediate prognosis mccRCC patients eligible for watchful waiting.

**Electronic supplementary material:**

The online version of this article (10.1007/s00259-019-04358-9) contains supplementary material, which is available to authorized users.

## Introduction

Renal cell carcinoma (RCC) accounts for 2% of all malignancies worldwide, with an estimated 403,262 new cases in 2018. Seventy percent have a clear cell component. Metastatic clear cell (mcc) RCC has a variable course, with a subgroup of patients showing slow disease progression. In those patients, it is safe to observe the course of disease in a period of so-called watchful waiting, avoiding unnecessary side-effects and costs of systemic treatment.

To identify patients eligible for watchful waiting, prognostic schemes such as the International Metastatic Database Consortium (IMDC) risk model have been used to differentiate between patients with a good, intermediate or poor prognosis [1, 2]. For staging mRCC, European Society of Medical Oncology (ESMO) guidelines mandate contrast-enhanced computed tomography (CT) of chest, abdomen and pelvis [3].

Previously, an international phase II study in mRCC patients eligible for watchful waiting showed that higher numbers of IMDC adverse risk factors (*p* = 0.0403) and higher numbers of metastatic disease organ sites (*p* = 0.0414) were associated with a shorter period of watchful waiting [4]. These results substantiate the clinical value of imaging, which may be further enhanced by molecular imaging with [^18^F]FDG or emerging radiopharmaceuticals targeting tumor-associated antigens like carbonic anhydrase IX (CAIX) to identify patients in need of urgent systemic or local therapy.

CAIX is over-expressed in 94% of ccRCC-tumors due to a mutational loss of Von Hippel Lindau protein [5–7]. Prognostic implications of immunohistochemically determined CAIX-expression are unequivocal [7–12]. In-vivo assessment of CAIX-expression can be performed with radiolabeled girentuximab (anti-CAIX antibody) PET-imaging. This technique visualizes primary and metastatic ccRCC lesions [13–15]. The value of [^18^F]FDG-PET/CT combined with CT in diagnosing and staging mRCC is not established; however, [^18^F]FDG-PET/CT may have prognostic value, with a positive scan being unfavourable [16, 17]. The IMaging PAtients for Cancer drug selecTion (IMPACT)-RCC study (ClinicalTrials.gov: NCT02228954) was designed to assess the added value of [^89^Zr]Zr-DFO-girentuximab-PET/CT and [^18^F]FDG-PET/CT at presentation in predicting the duration of watchful waiting in patients with good or intermediate prognosis mccRCC.

Here, we report the lesion detection of [^89^Zr]Zr-DFO-girentuximab-PET/CT and [^18^F]FDG-PET/CT in mccRCC in addition to CT. We determined the lesion detection yield of the three modalities, assessed inter-observer agreement in [^89^Zr]Zr-DFO-girentuximab-uptake interpretation, and investigated determinants of quantitative [^89^Zr]Zr-DFO-girentuximab and [^18^F]FDG-uptake.

## Materials and methods

### Patients

In this prospective multicenter cohort study, patients aged 18 years and older with histologically or cytologically proven RCC with a clear cell component, recently (<6 months) diagnosed metastases, and a good or intermediate prognosis according to IMDC score [1], were enrolled in the IMPACT-RCC study conducted at four Dutch academic medical centers. A period of watchful waiting for 2 months was considered optional according to treating medical oncologist. Patients who received any previous systemic treatment for RCC in any setting were excluded, but previous radiotherapy and surgery (nephrectomy or metastasectomy) was permitted. Furthermore, patients were excluded in the presence of untreated central nervous system metastases or symptomatic intra-cerebral metastases, pregnant or breast feeding women. Only patients without prior systemic treatment were enrolled, therefore the IMDC criteria ‘time from diagnosis to treatment <1 year’ was adapted into ‘time from primary diagnosis to diagnosis of metastatic disease <1 year’. Watchful waiting was terminated if radiological disease progression was established, in combination with a clinical need to start systemic treatment.

### Patient imaging

Patients underwent CT, [^18^F]FDG and [^89^Zr]Zr-DFO-girentuximab-PET/CT at the start of the watchful waiting period. Further details on the imaging modalities (acquisition and reconstruction protocols) and the conjugation, radiolabeling and quality control of [^89^Zr]Zr-DFO-girentuximab are provided in the Supplements.

### Image assessment

All CT and [^18^F]FDG-PET/CT scans were reported according to standard clinical practice by an experienced local radiologist and nuclear physician, respectively. The assessment of CT lesions was performed according to RECIST 1.1 [18]; however, to ensure measurements and documentation of all lesions including non-target lesions of ≥10 mm, CT scans were independently revised by one or two experienced radiologists (E.H.A; T.C.K.). The [^89^Zr]Zr-DFO-girentuximab PET/CTs were assessed in a central reviewing system to ensure true lesion detection and reproducible inter-observer agreement.

All [^89^Zr]Zr-DFO-girentuximab PET/CTs were assessed by three expert nuclear physicians independently (W.O.; A.H.B.; O.H.) through online central reviewing system designed by CTMM TRaIT. The three reports were harmonized to one final report by one designated reviewer. In case of different findings, a meeting was organized to reach consensus. The treating physician was blinded for the results of either PET/CT; however, for patient safety reasons, the nuclear physician was allowed to communicate findings that required (local) interventions (e.g. brain metastases).

A tumor lesion was defined visually positive based on anatomical substrate on low-dose CT in combination with [^18^F]-FDG and/or [^89^Zr]Zr-DFO-girentuximab-uptake, or solely on prominent, non-physiological antibody-uptake. Quantification of positive lesions as defined by evaluation reports for [^18^F]FDG and [^89^Zr]Zr-DFO-girentuximab-PET/CT was performed by drawing regions-of-interest using Inveon Research Workplace software (IRW, version 4.1). The maximum and mean standardized uptake values (SUV) were calculated. SUV_max_ was used for tumor tracer-uptake; SUV_mean_ for measuring uptake in healthy organs and blood pool.

### Statistical analysis

To compare the agreement in individual lesion detection between observers, we used dependent pair wise or multi-observers kappa-coefficients with the delta method [19]. Lesion detection rates per imaging modality and combined imaging modalities (CT combined with PET/CT) were estimated and compared (by Wald tests) using mixed effect logistic regression models accounting for within patient and lesion-clustering by random intercepts. We evaluated lesion detection rates overall and according to organ sites. Furthermore, we compared the median number of affected organ sites across patients assessed by CT only, or in conjunction with either PET/CT using Wilcoxon signed rank tests.

To assess biodistribution of [^89^Zr]Zr-DFO-girentuximab, we estimated the average SUV_mean_ per organ and compared variability within and between patients (one-sample T-test). SUV_max_ was evaluated using descriptive methods besides mixed effects linear regression models, taking within patient clustering into account as random intercepts (using intra-class correlation coefficient (ICC) to estimate variation in uptake due to between-patient heterogeneity). These models were also used to assess determinants of tracer-uptake (introduced as fixed effects and compared by Wald tests). SUV_max_ was natural log-transformed to obtain appropriate model fit, resulting in geometric means or percent changes in SUV_max_ as interpretation of fixed effects. We fitted these models under restricted maximum likelihood using Satterthwaite approximations to degrees of freedom. We used the marginal R^2^ to estimate the variance in tracer-uptake explained by the fixed effects of these models [20], then fitted under maximum likelihood.

We report estimates with 95% confidence intervals (CI), and statistical tests were two-sided with threshold for significance of 5%, without adjusting for multiple testing. Analyses were performed in R (version 3.2.1), particularly using libraries multi-agree (version 2.1), lme4 (version1.1-11), lmerTest (version2.0-20), and MuMIn (version1.10.0).

## Results

### Patients

From February 2015 until March 2018, 42 mccRCC patients were included. All patients had a histopathological diagnosis of the primary tumor, either through (partial) nephrectomy or biopsy in 36 and six patients, respectively. A total of 14 patients had a favourable prognosis. Of the remaining 28 patients, 13 had a predicted intermediate prognosis with one risk factor and 15 patients with two risk factors. This was primarily due to the diagnosis of metastases <1 year after the primary diagnosis (80%) and/or the presence of anaemia (51%). There was no correlation between histology (e.g. mixed vs. pure clear cell) and the estimated prognosis according to IMDC.

All patients without a previous nephrectomy had an estimated intermediate prognosis. In total 57% of all patients presented with metachronous metastases at a median interval of 0.7 (range 0–15) months between primary diagnosis and first metastasis. One patient presented with only sub-centimeter indeterminate lung lesions; therefore, lesions were not included in the analyses. Five others had a negative [^18^F]FDG-PET/CT, of whom one plus two other patients had a negative [^89^Zr]Zr-DFO-girentuximab-PET/CT. In two patients, the [^18^F]FDG-PET/CT and/or [^89^Zr]Zr-DFO-girentuximab-PET/CT revealed brain metastases warranting local treatment with stereotactic radiotherapy and temporary treatment with corticosteroids.

**Fig. 1:**
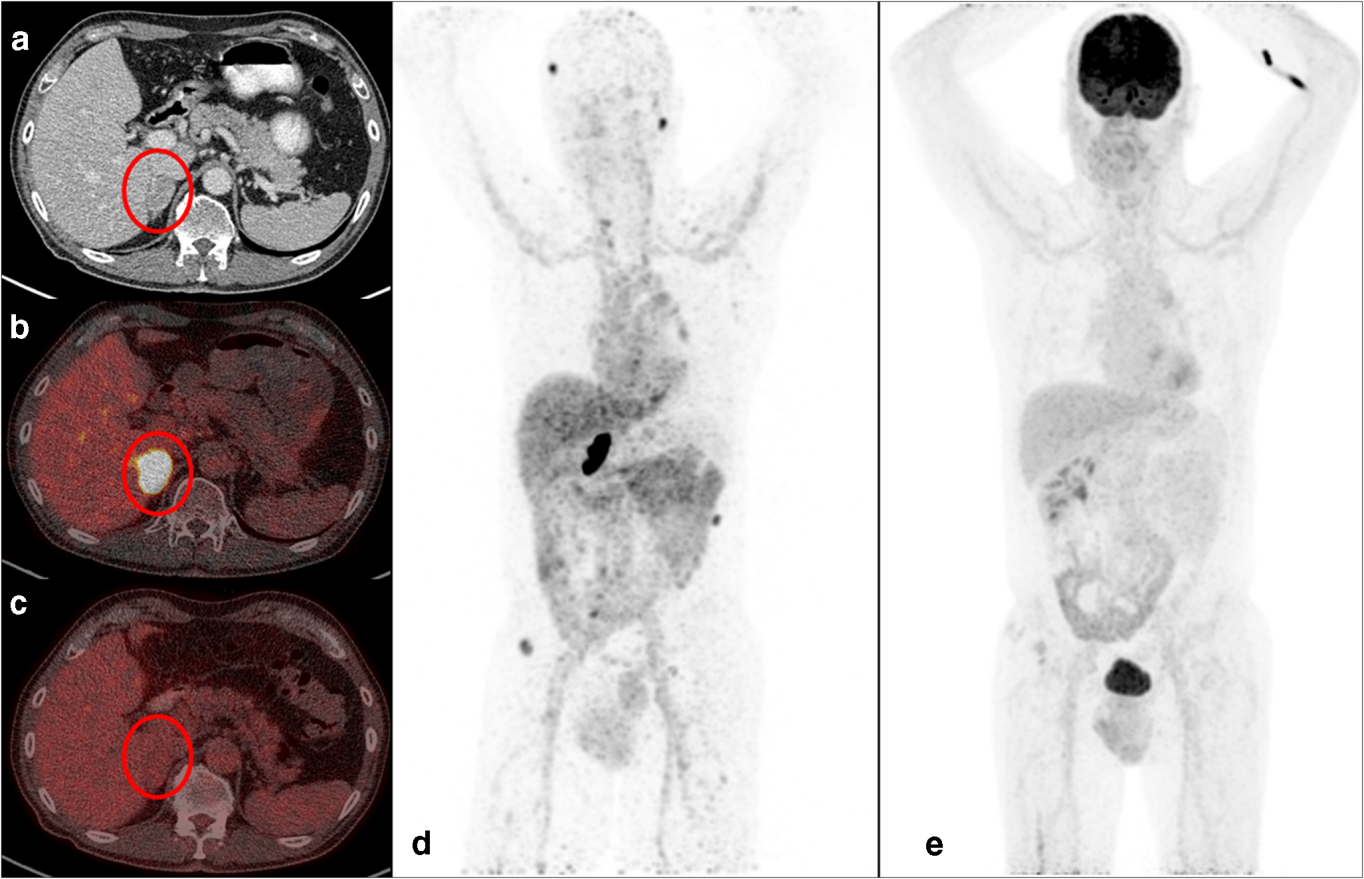
On the left are transversal sections of one patient of CT, [^89^Zr]Zr-DFO-girentuximab and [^18^F]FDG-PET/CT. The *red circle* represents an adrenal gland lesion in a patient as visualized by CT (**a**), [^89^Zr]Zr-DFO-girentuximab (**b**) and [^18^F]FDG-PET/CT (**c**), respectively. On the right, MIP images of [^89^Zr]Zr-DFO-girentuximab (**d**) and [^18^F]FDG-PET/CT (**e**) are presented

Patient characteristics are shown in Table 1, imaging examples are shown in Fig. 1.

**Table 1:** Patient demographics and clinical characteristics

Parameter	Patients (*n* = 42)
Sex	
Male	31 (74%)
Female	11 (26%)
Age (years)	
Median (range)	66.1 (44–86)
Nephrectomy	
Yes	36 (86%)
No	6 (14%)
Histology	
Pure clear cell	32 (76%)
Mixed	10 (24%)
Location of first metastases^a^	
Lung^b^	22 (52%)
Adrenal gland	4 (10%)
Lymph node	9 (21%)
Bone	2 (5%)
Kidney	2 (5%)
Other^c^	3 (7%)
Time from diagnosis to first metastases (median 0.7; range 0–15 months)	
<1 year	23 (55%)
≥1 year	19 (45%)
IMDC risk factors	
0 (favorable)	14 (33%)
1 (intermediate)	13 (31%)
2 (intermediate)	15 (36%)

^a^ 57% presented with synchronous metastases

^b^ Five patients had lung-only disease (based on CT only).

^c^ Two patients presented with soft tissue metastases, one patient with multiple involved organ sites

### Lesion detection rates of CT, [^18^F]FDG and [^89^Zr]Zr-DFO-girentuximab-PET/CT

A total of 449 lesions were identified by at least one modality (median per patient, 7; ICR 4.25–12.75). Lesions were located in lung (42%), lymph nodes (22%), bone (10%), soft tissue (8%), adrenal gland (6%), kidney (4%), pancreas (4%) or elsewhere (4%).

Lesion detection rates differed across modalities: 56% was visualized by CT (95%CI 50–62). [^18^F]FDG-PET/CT detected 59% (95%CI 53–65; *p* = 0.37). [^89^Zr]Zr-DFO-girentuximab-PET/CT visualized 70% (95%CI 64–75), which was more than CT alone (*p* < 0.001) or [^18^F]FDG-PET/CT alone (*p* < 0.005). Nine of 449 (2%) lesions were outside the field of view of CT (brain *n* = 2; lymph nodes in the neck *n* = 4, bone (extremities) *n* = 3). Agreement in detecting lesions between modalities was poor; kappa’s −0.12 (95%CI −0.25;0.01), −0.00 (95%CI −0.13;0.12), and 0.20 (95%CI 0.02;0.37) for CT and [^89^Zr]Zr-DFO-girentuximab-PET/CT, CT and [^18^F]FDG-PET/CT, and [^89^Zr]Zr-DFO-girentuximab-PET/CT and [^18^F]FDG-PET/CT, respectively.

Agreement between two radiologists in identifying lesions on CT was moderate (kappa 0.51; 95%CI 0.42–0.59), and substantial for three nuclear physicians assessing [^89^Zr]Zr-DFO-girentuximab-PET/CTs (kappa 0.71; 95%CI 0.60–0.82).

### Combination of modalities for lesion detection

**Fig. 2:**
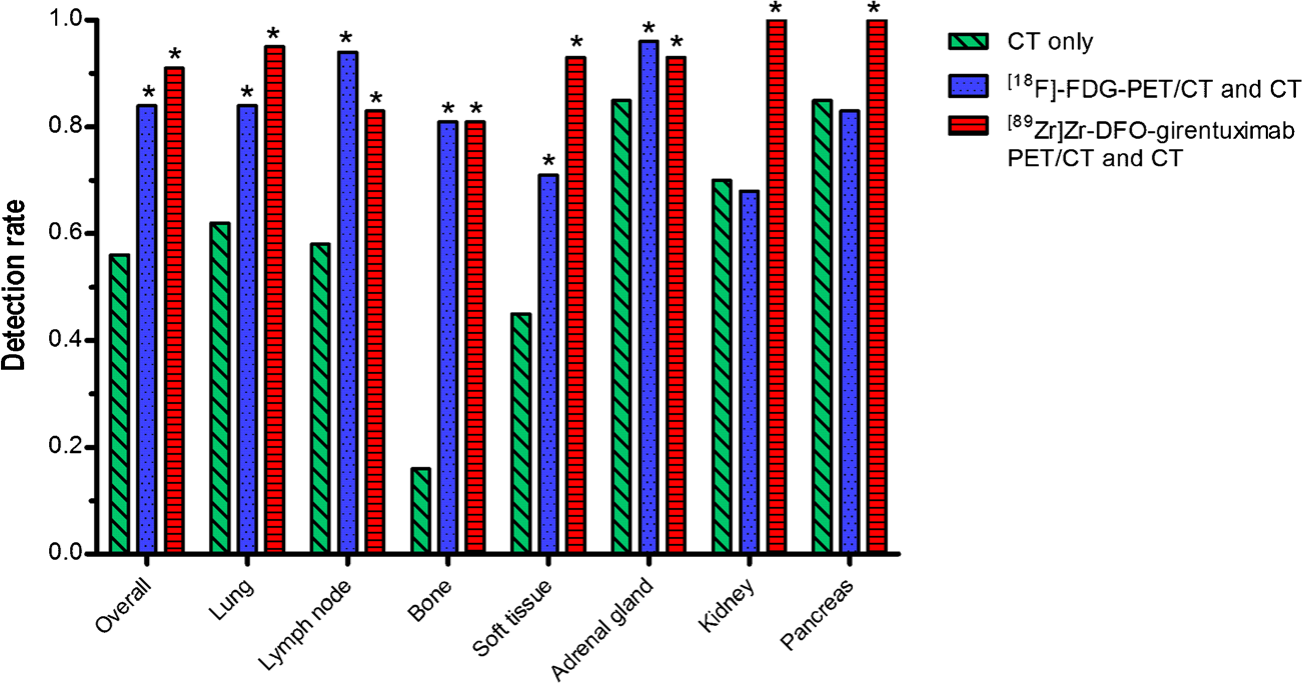
Lesion detection per imaging modality and per organ. Concordant pairs were lesions that were visualized on all three modalities. Nine PET detected lesions were outside the field of view of CT. *p < 0.001 compared to CT only

With the addition of [^89^Zr]Zr-DFO-girentuximab-PET/CT and [^18^F]FDG-PET/CT, lesion detection by CT alone increased from 56% to 91% (95%CI 87–94) and 84% (95%CI 79–88), respectively. Improved lesion detection rate was apparent for all organ sites (Fig. 2). The lesion detection of CT-[^89^Zr]Zr-DFO-girentuximab-PET/CT was better than CT-[^18^F]FDG-PET/CT (*p* < 0.005). Largest improvement was seen in the number of bone lesions, with 81% of all bone lesions detected by both [^89^Zr]Zr-DFO-girentuximab-PET/CT and CT as well as [^18^F]FDG-PET/CT with CT, compared to 16% by CT alone (*p* < 0.001). More lung lesions were detected by CT-[^89^Zr]Zr-DFO-girentuximab-PET/CT compared to CT-[^18^F]FDG-PET/CT [95% (95%CI 91–98)] versus 84% (95%CI 76–89; *p* < 0.001). Lesion detection approached 100% in pancreas and kidney with combined CT and [^89^Zr]Zr-DFO-girentuximab-PET/CT. Conversely, detecting enlarged lymph nodes was better with combined [^18^F]FDG-PET/CT and CT [94% (95%CI 88–97)], compared to [^89^Zr]Zr-DFO-girentuximab-PET/CT and CT [83% (95%CI 73–89, *p* < 0.05)].

### Assessment of affected organ sites

The median number of affected organ sites increased with the addition of [^89^Zr]Zr-DFO-girentuximab-PET/CT or [^18^F]FDG-PET/CT compared to CT alone in 27 patients (median increased from 2 to 3, range 1–7 (*p* < 0.005). [^89^Zr]Zr-DFO-girentuximab-PET/CT and [^18^F]FDG-PET/CT performed similarly (Table 2). Patients were categorized according to the location of their lesions (e.g. lung only; other organ(s) only and both lung and other organs). With the addition of both PET/CTs, two patients were re-categorized from lung only into ‘both lung and other organs’ based on the additional detected lymph node and bone lesions (Table 1).

**Table 2:** The number of affected organ sites per patient per imaging modality (combination)

Number of organ sites with metastases per patient			
CT only (median 2)		[^18^F]FDG-PET/CT and CT (median 3)*	[^89^Zr]-DFO-girentuximab-PET/CT and CT (median 3)*
0	2.4%	–	–
1	33.3%	23.8%	23.8%
2	35.7%	21.4%	26.1%
3	21.4%	38.1%	30.9%
4	7.1%	14.2%	11.9%
5	–	2.4%	4.8%
7	–	–	2.4%

*Significant increase of the median number of organ sites compared to CT alone (*p* < 0.005)

### Quantitative analysis of [^89^Zr]Zr-DFO-girentuximab and [^18^F]FDG-uptake

In normal tissues the highest [^89^Zr]Zr-DFO-girentuximab-uptake was observed in healthy liver (geometric mean SUV_mean_ 6.7 (95%CI 6.4–7.3), lowest in healthy lung (geometric mean SUV_mean_ 1.1 (95%CI 0.8–1.2) (*p* < 0.05). The physiological biodistribution of [^89^Zr]Zr-DFO-girentuximab is illustrated in the Supplements.

The overall geometric mean [^89^Zr]Zr-DFO-girentuximab SUV_max_ in lesions was 15.5 (95%CI 12.5–19.2), and 4.4 (95%CI 3.8–5.1) for [^18^F]FDG. Tracer uptake was higher in lesions with a CT diameter > 15 mm, compared to smaller lesions (geometric mean SUV_max_ 23.9 (95%CI 19.0–30.0) and 5.8 (95%CI 5.0–6.8 for ^89^Zr-DFO-girentuximab and geometric mean 11.6 (95%CI 9.3–14.5) and 3.5 (95%CI 3.0–4.1) for [^18^F]FDG). Based on expert opinion, for further analyses of tracer uptake a cut-off of ≥15 mm in diameter on CT was chosen to avoid partial volume effects thwarting proper quantification (leaving 95 lesions in 26 patients for [^89^Zr]Zr-DFO-girentuximab, and 93 lesions in 29 patients for [^18^F]FDG).

**Fig. 3:**
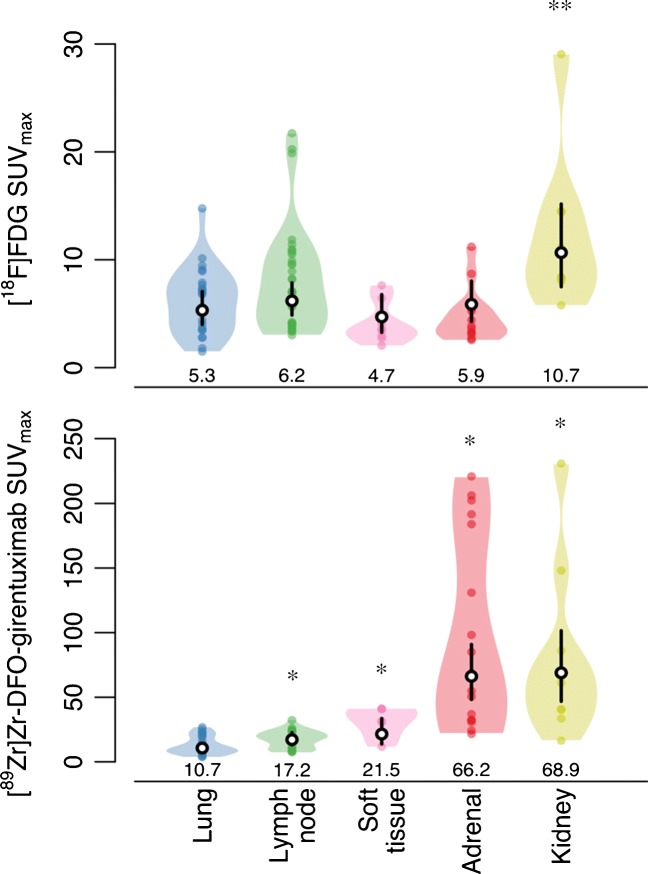
A Violin plot of actual distribution of [^89^Zr]Zr-DFO-girentuximab and [^18^F]FDG SUV_max_ in tumor lesions per organ site. *Black vertical lines* are 95% CIs of geometric mean SUVmax, *white dots within black lines* and values are the actual geometric means; *coloured dots* are individual metastases. The locations represent organ sites with at least five suspect lesions. *Compared to lung lesions, a difference was seen in the height of [^89^Zr]Zr-DFO-girentuximab SUV_max_ values of lymph node, soft tissue, adrenal gland and kidney lesions (*p* < 0.05). **The height of [^18^F]FDG SUV_max_ values of kidney lesions was significantly higher compared to soft tissue lesions (*p* < 0.05)

The [^89^Zr]Zr-DFO-girentuximab SUV_max_ varied greatly, ranging from 3.8 to 230.8, with a median-fold difference of 2.8 (range 1.2–15.3) per patient. Inter-patient heterogeneity accounted for 41% of variation in [^89^Zr]Zr-DFO-girentuximab SUV_max_, and 53% for [^18^F]FDG SUV_max_ (i.e. ICC of 0.41 and 0.53, respectively). Highest [^89^Zr]Zr-DFO-girentuximab-uptake was seen in kidney and adrenal gland lesions (median SUV_max_ 61.1 and 69.9, respectively) and lowest in lung lesions (median SUV_max_ 9.4) (Fig. 3). Two out of six patients without prior nephrectomy showed highest [^89^Zr]Zr-DFO-girentuximab-uptake at primary site (SUV_max_ 70.52 and 40.48), compared to synchronous metastatic sites.

### Determinants of tracer-uptake

[^18^F]FDG-uptake was not related to [^89^Zr]Zr-DFO-girentuximab-uptake (*p* = 0.29). Univariable-analysis showed a strong relation of tracer-uptake to lesion location (*p* < 0.005; explaining 61% and 12% of the variation in [^89^Zr]Zr-DFO-girentuximab and [^18^F]FDG SUV_max_). Largest measured CT lesion diameter was associated with tracer-uptake (*p* < 0.001, explaining 13% and 16% of the variation in [^89^Zr]Zr-DFO-girentuximab and [^18^F]FDG SUV_max_), with [^89^Zr]Zr-DFO-girentuximab SUV_max_ increasing on average 59% (95%CI 25–102) and [^18^F]FDG SUV_max_ 33% (95%CI 14–54) per doubling diameter.

In multivariable analysis, mutual adjustment for location, size, and uptake of the other tracer did not substantially alter the correlation between tracer uptake and location. Size and [^89^Zr]Zr-DFO-girentuximab SUV_max_ were no longer related [estimated average change in uptake of 3% (95%CI −17 to 28) per doubling size], whereas the relation between size and [^18^F]FDG SUV_max_ did not change substantially [estimated change in uptake of 32% (95%CI 11–58) per doubling size]. Thus, [^89^Zr]Zr-DFO-girentuximab-uptake was mainly dependent on lesion location, and little affected by size and uptake of the [^18^F]FDG (which together explained 63% compared to 61% by location alone).

## Discussion

This lesion detection analysis in newly diagnosed mccRCC patients with a good or intermediate prognosis according to IMDC criteria and eligible for watchful waiting, demonstrates that addition of [^89^Zr]Zr-DFO-girentuximab-PET/CT to CT in the diagnostic work-up increases overall detection of mccRCC lesions from 56% to 91%. The number of detected bone- and soft tissue lesions increased, and all renal and pancreatic lesions were detected with this combination of modalities. In this patient selection, [^89^Zr]Zr-DFO-girentuximab-PET/CT and CT resulted in the detection of more mccRCC lesions than [^18^F]FDG-PET/CT and CT (*p* = 0.006). Considering the expected proportion of false-positive lymph node lesions on [^18^F]FDG-PET/CT due to [^18^F]FDG uptake in reactive (mostly mediastinal) lymph nodes, this difference in detection rate is in favour of [^89^Zr]Zr-DFO-girentuximab-PET/CT and CT.

A patient’s prognosis is estimated based on the number of involved organs on CT, total disease burden and period of watchful waiting, rather than the number of lesions [4, 21]. In our study population 33% of the patients present with a predicted good prognosis mRCC and 43% of patients with synchronous metastases. This is comparable to previous datasets and reflects daily clinical practice [4]. Patients with lung-only metastases are thought to have a better prognosis than other involved organ sites such as liver and bone [22, 23]. In our study population, based on CT only, seven patients (17%) presented with lung-only metastases. This number was revised after the addition of PET/CT because of the detection of additional bone and lymph node lesions by PET/CT in two patients. Furthermore, two patients were diagnosed with brain metastases by [^89^Zr]Zr-DFO-girentuximab PET/CT that required local treatment.

Overall, the median number of two involved organs per patient as determined by CT alone increased to three per patient with the addition of PET/CT (range 1–7; *p* < 0.005), even without adjusting for the limited CT field-of-view. This is largely attributed to the detection of more soft-tissue and bone lesions, a well-known limitation of CT due to less soft tissue contrast and the limited ability to detect (non-lytic) bone lesions. This limited increase in the number of involved organ sites with the addition of PET/CT questions its additional value, since solely an increase in detection lesions will not lead to the implementation of [^89^Zr]Zr-DFO-girentuximab or [^18^F]FDG-PET/CT to our standard work-up. However, [^89^Zr]Zr-DFO-girentuximab and [^18^F]FDG PET/CT findings were clinical and possibly prognostic relevant in at least 10% of patients and warrants further investigation.

The interpretation of involved organ sites in all three modalities was challenging, especially considering the limitation of each modality. For example, spatial resolution is lower with PET/CT compared to CT, resulting in a partial volume effect affecting small (<2 cm), low-contrast lesions both visually and quantitatively [24]. CT can detect sub-centimeter or indeterminate pulmonary nodules and lymph nodes, although distinguishing nonspecific from small metastatic lesions with CT is notoriously difficult. Based on studies of pulmonary metastases in RCC and RECIST 1.1 criteria, we used a diameter cut-off of 10 mm and in lymph nodes 15 mm to prevent overestimating of the number of detected lesions [25, 26]. This ultimately reduced the number of (small) lung and lymph node lesions detected by CT, thereby underestimating the overall lesion detection by CT.

All lesions visible on either CT, [^89^Zr]Zr-DFO-girentuximab or [^18^F]FDG-PET/CT were defined as metastases, which introduces potential bias and a risk of possible false-positive. Despite the high specificity of [^89^Zr]Zr-DFO-girentuximab to visualize primary and metastatic ccRCC-lesions expressing CAIX [5, 13–15, 27] and the careful assessment of [^89^Zr]Zr-DFO-girentuximab PET/CT by three independent nuclear physicians with a fairly good agreement (kappa 0.71;95%CI:0.60–0.82), our results are limited by the lack of histological confirmation of the detected lesions. Lesions not visible on CT but only [^89^Zr]Zr-DFO-girentuximab and [^18^F]FDG PET/CT could be false-positive lesions. Alternatively, the tracer-uptake may resemble a new tumor lesion that is not yet visible on CT due to a dedifferentiated state with a different metabolic state and could become apparent in a period of follow-up. Finally, false negative lesions could be present as well; however, with the available data we cannot draw any conclusions on this.

Interestingly, [^18^F]FDG and [^89^Zr]Zr-DFO-girentuximab-uptake strongly depend on the organ where the lesion is localized, which is also previously described for [^89^Zr]Zr-bevacizumab uptake in mccRCC [28]. Overall, highest [^18^F]FDG and [^89^Zr]Zr-DFO-girentuximab SUV_max_ values were visualized in metastases of the adrenal gland and the kidney. In the six patients without previous nephrectomy, the highest SUV_max_ value was measured in the metastatic lesions and not in the primary tumor. Organ-specific characteristics influence [^89^Zr]Zr-DFO-girentuximab-uptake, e.g. presence of stromal and immune cells, stroma and/or vasculature affecting perfusion. This is illustrated by the notably high SUV_max_ values of [89Zr]Zr-DFO-girentuximab uptake in adrenal gland lesions as compared to other lesion sites, e.g. lung (median SUV_max_ 69.9 and 9.4, respectively).

Depending on the clinical question, both [^89^Zr]Zr-DFO-girentuximab and [^18^F]FDG-PET/CT are valuable as additional imaging techniques by visualizing whole-body mccRCC lesions where the combination of [^89^Zr]Zr-DFO-girentuximab with CT increases the total number of detected lesions most and supports the role of [^89^Zr]Zr-DFO-girentuximab-PET/CT in the early detection of mccRCC lesions [27]. Furthermore, the quantification of tracer-uptake in both PET-imaging modalities offers a better understanding of the heterogenic study population [4]. Combining anatomical imaging techniques with functional imaging techniques targeting glucose metabolism and CAIX expression offers a better representation of the heterogeneity by visualizing whole body tumor nature and active metabolic processes (e.g. glycolysis, GLUT-1-expression) [8].

Upon completion of the follow-up data of all patients included in the IMPACT-RCC study, we will analyze whether [^89^Zr]Zr-DFO-girentuximab and [^18^F]FDG-PET/CT contributes to a better prediction of the course of disease during watchful waiting in good and intermediate prognosis mccRCC patients.

## Conclusions

The addition of [^89^Zr]Zr-DFO-girentuximab-PET/CT and [^18^F]FDG-PET/CT to CT increases lesion detection compared to CT alone in newly diagnosed good and intermediate prognosis mccRCC patients eligible for watchful waiting. The quantitative analyses of ^89^Zr-DFO-girentuximab and [^18^F]FDG-uptake can be relevant in clinical practice, as site-specific heterogeneity may require a different treatment approach.

## Electronic supplementary material

ESM 1 (DOCX 296 kb)
