# Supplementary material for: Lesion detection by [89Zr]Zr-DFO-girentuximab and [18F]FDG-PET/CT in patients with newly diagnosed metastatic renal cell carcinoma
Source: Eur J Nucl Med Mol Imaging. 2019 Jun 6;46(9):1931–9. doi: 10.1007/s00259-019-04358-9 (PMC6647180; doi:10.1007/s00259-019-04358-9)
Supplement: Supplementary file 1 — (DOCX 296 kb) [file 259_2019_4358_MOESM1_ESM.docx]

**Supplements**

**Participating centers**

Patients were enrolled in the Radboud university medical center (UMC), UMC Groningen, Amsterdam UMC and Erasmus medical center (MC).

**Patient imaging**

**CT-scan**

The CT acquisition and reconstructions were performed according to local protocols for Canon Aquilion One GENESIS Edition (RadboudUMC), Siemens Force/Flash (UMC Groningen) scanner, Discovery CT750 (Amsterdam UMC) and Siemens Somatom (Erasmus MC).

Acquisition protocols were as follows;100 or 120 kV protocol (automatic exposure control (AEC) with standard deviation (SD) of 15), with auto mA 120-500, noise index of 25, at a rotation speed of 0.275-0.5 sec. Scan range included chest, abdomen and pelvis. Reconstruction was performed by the Canon Aquilion scanner using adaptive iterative dose reduction 3dimensional enhance (AIDR 3Denh) in combination with FC08 filter to create axial in 1mm/0.8mm and axial, coronal and sagital in 5mm/4mm slices and FC86 filter to create axial in 1mm/0.8mm and axial in 5mm/4mm and 10/3 MIP axial. Images from the Flash/Force scanner were reconstructed using SAFIRE iterative reconstructions programme 2 to create 1mm slices with an increment of 0.7mm for chest reconstructions and 2mm slices with an increment of 1,5mm in slices of the abdomen and pelvis. CT images of the Discovery CT750 were reconstructed using the adaptive statistical iterative reconstruction (ASIR) algorithm at 60-70% to create 0.625 mm axial and coronal slices of the chest and 3mm slices of the abdomen and pelvis. SAFIRE iterative reconstructions were also used to reconstruct images from the Siemens Somatom to create either axial in 3mm/3mm and 3mm/2mm coronal/saggital or axial in 1mm/0.8mm

Image analysis was performed on the venous phase scans after intravenous injection of iodinated contrast at 100-150 ml/kg body weight with bolus tracking at a delay of 30-80 sec (chest - abdomen) and maximal slice thickness of 5.0 mm.

**Patient imaging – PET/CT**

[^18^F]FDG-PET/CT was performed according to European Association of Nuclear Medicine (EANM) guidelines version 1.0 [26] and the [^89^Zr]Zr-imaging procedure was harmonized between participating, EARL-accredited centers (PET/CT-systems)[27]. Patients underwent [^89^Zr]Zr-DFO-girentuximab-PET/CT 4 days after intravenous (IV) injection of 37 MBq [^89^Zr]Zr-DFO-girentuximab (protein dose 5 mg). For both PET scans, patients were scanned from the head to upper thigh in up to 6 consecutive bed positions, during 5 minutes for each bed position with a 64-slice PET/CT camera (Biograph mCT, Siemens in RadboudUMC, UMC Groningen and Erasmus MC; Gemini TF or Ingenuity TF, Philips in the Amsterdam UMC). All data were corrected for dead time, scatter, randoms, decay and tissue attenuation, with a final reconstruction resolution of 7 mm.

**Conjugation, radiolabeling and quality control of [^89^Zr]Zr-DFO-girentuximab**

[^89^Zr]Zr-girentuximab was produced under good manufacturing practice (GMP) requirements with ≥95% radiochemical purity.

To allow for [^89^Zr] labeling, Girentuximab (Wilex AG, Munich, Germany) was conjugated with the chelator N-succinyldesferrioxamine-B-tetrafluorphenol (N-SucDf-TFP) (obtained from VU University Medical Center, Amsterdam, the Netherlands). Previously, the [^89^Zr] labeling of girentuximab has only been described for preclinical studies [28]. After conjugation the intermediate girentuximab-desferal was purified using gel permeation columns (PD10, GE Healthcare Life sciences, Eindhoven, The Netherlands). After dilution to a concentration of 2 mg/ml it was filtered through 0.2 μm Millex GV filter and dispensed.

Radiolabeling of DFO-girentuximab with [^89^Zr] was performed under GMP conditions at RadboudUMC and transported to participating centers. The final formulation of the radiolabeled product contains a total concentration of girentuximab ([^89^Zr]Zr-DFO-girentuximab + unlabeled girentuximab) of 5 mg/10 ml. The unlabeled antibody is added to prevent possible hepatic uptake of the radiolabeled mAb. The final product contains 37 MBq [^89^Zr]Zr-DFO-girentuximab at the time of injection. The radiochemical purity (thin layer chromatography, high performance liquid chromatography), was ≥ 95%, while immunoreactive fraction as assessed by a cell binding assay exceeded 80%[29].

**
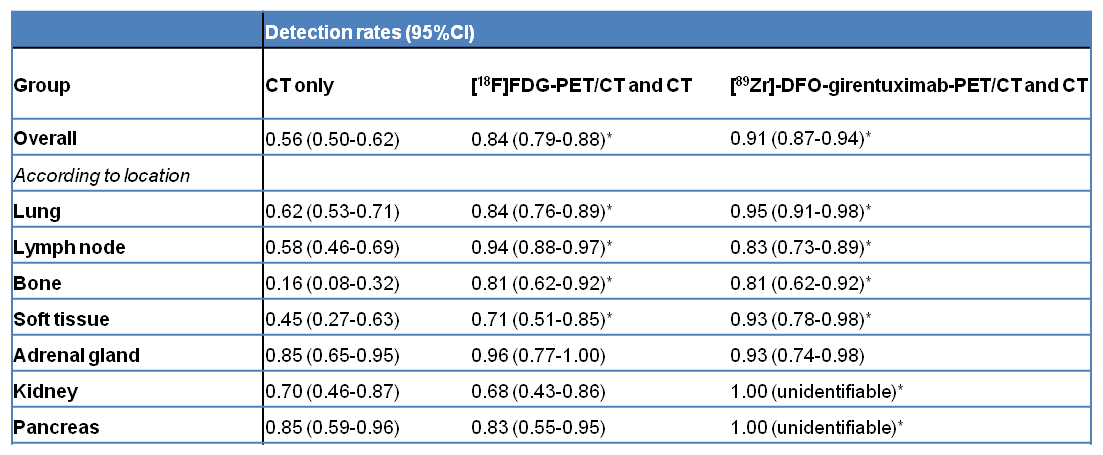
**

**Table 1.** Lesion detection per imaging modality and per organ**.**

Concordant pairs were lesions that were visualized on all 3 modalities. 9 PET detected lesions were outside the field of view of CT. **p* < 0.001 compared to CT only.

**

**

**Fig. 1** Biodistribution of [^89^Zr]Zr-DFO-girentuximab in normal organs. Black vertical lines are 95% CIs of geometric mean SUVmean, centered black lines represent the actual geometric means; coloured dots are individual measurements. The SUV_mean_ geometric mean was significantly higher than all other healthy organs (*p* < 0.05). (*n*=41 per organ site; except for the adrenal gland (*n*=27).
